# Morphological analysis and cytotoxicity of acrylamide on SPC212 human mesothelioma cells: Do low doses induce proliferation, while high doses cause toxicity?

**DOI:** 10.1111/jcmm.70190

**Published:** 2024-11-08

**Authors:** Sedat Kacar, Ozlem Tomsuk

**Affiliations:** ^1^ Department of Histology and Embryology, Faculty of Medicine Eskisehir Osmangazi University Eskisehir Turkey; ^2^ Division of Pulmonary, Critical Care, Sleep and Occupational Medicine, Department of Medicine Indiana University Indianapolis Indiana USA; ^3^ Department of Biotechnology and Biosafety, Graduate School of Natural and Applied Sciences Eskisehir Osmangazi University Eskisehir Turkey; ^4^ Cellular Therapy and Stem Cell Production Application and Research Center (ESTEM) Eskişehir Osmangazi University Eskişehir Turkey

**Keywords:** acrylamide toxicity, apoptosis, Bax, Bcl‐2, H‐E, immunocytochemistry, MTT, neutral red, PCNA, proliferation, SPC212 human mesothelioma cells

## Abstract

Acrylamide is broadly utilized in numerous areas with different purposes including being an additive, flocculating, sealing, dry strength improver and polymerizing agent, and so forth. Furthermore, it forms in certain food products at high temperatures. It poses serious hazard since its readily water‐soluble and very reactive nature. Besides in vivo studies, several in vitro studies with various cell lines are carried out to evaluate its toxicity. However, of these cell line studies, there are no mesothelium or mesothelioma cell lines. To fill this lacuna, we aimed at examining various dose range of acrylamide on SPC212 human mesothelioma cell line. First, we executed MTT and neutral red cytotoxicity tests and ascertained IC50 dose. Next, we performed inverted, light (haematoxylin–eosin and May Grünwald), fluorescent (DAPI) and confocal microscope (AO/EB) analyses as well as immunohistochemistry for Bax, Bcl‐2 and PCNA proteins. As a result, we found IC50 of acrylamide at 2.65 mM. Starting from 3.13 mM of acrylamide dose, a deep decrease in cell proliferation was observed. Particularly in MTT assay, a proliferative action of acrylamide was detected at 0.39 and 0.78 mM, supported with inverted microscope images. In light microscope analysis, several cellular degenerations, including condensed and kidney‐shaped nucleus were evident. In AO/EB staining, cells with apoptotic characteristics augmented dose‐dependently, being upheld by a parallel uptick in Bax and a dimunition in Bcl‐2 staining. Besides, PCNA decreased at IC50 dose of acrylamide. This is the acrylamide‐associated first study conducted on SPC212 mesothelioma cells encompassing advanced morphological analysis. We believe this study to be an incentive for future studies.

## 1 INTRODUCTION

Acrylamide (C₃H₅NO) is a reactive monomer that has a pristine white hue, is characterized by a crystalline nature and devoid of any discernible odour. Its chemical constitution comprises a polar amide group and a vinyl functionality, rendering it amenable to acrylamide polymerization. This compound, a reactive α, β‐carbonyl unsaturated entity, is typically synthesized through the hydration of acrylonitrile via sulfuric acid monohydrate at elevated temperatures of 90 to 100°C. Commercially available for industrial applications since 1950, acrylamide has found widespread utility in various sectors. It is broadly utilized in numerous areas with different purposes including being an additive, flocculating, sealing, dry strength improver and polymerizing agent, and so forth.^1^

In the literature search engines of Web of Knowledge, PubMed, ScienceDirect and Scopus, the oldest study associated with acrylamide toxicity dates back to the year of 1956.^2^ In 1994 International Agency For Research on Cancer categorized acrylamide among Group 2A substances, which are probably carcinogenic to humans.^3^ In 1997, the workers are exposed to sealants containing acrylamide for 2 months while constructing a tunnel for railways in Sweden. They displayed some peripheral nervous system‐related problems. Their haemoglobin adducts of acrylamide were quantified and revealed to be directly proportional to these problems.^4^ In 2002, Tareke et al. revealed that acrylamide forms in food by Maillard reaction upon high‐temperature process, which was the breakthrough for perceiving the importance of acrylamide.^5^

The studies of acrylamide toxicity have been investigated almost for 65 years on, setting off from nervous system to reproductive system, and then shifted to the other organ and systems. This increase in toxic relevance of acrylamide is not only due to its existence in food but also being readily soluble in water and very reactive, therefore having to capability to reach every part of the body by bloodstream once ingested. Acrylamide is an electrophile and has a high tendency to react with DNA, RNA and sulfhydryl containing macromolecules. And it poses far more hazard to kids than the rest of the population according to food mass/body weight estimation.^6^ Nowadays, acrylamide was also claimed to possess endocrine disrupting potential,^7^ although this hypothesis was not substantiated previously.^8^

Besides in vivo studies,^9^, ^10^ several in vitro studies are carried out to evaluate its toxicity. Of these, lung,^11^ fibroblast,^12^ neuroblastoma,^13^ liver,^14^ glioblastoma,^15^ pheochromocytoma cells,^16^ and so forth have been studied so far. However, no in vitro acrylamide toxicity study was performed in mesothelium or mesothelioma cells, albeit 4 in vivo studies correlating acrylamide with mesothelioma cancers.^8^, ^17^, ^18^, ^19^ To summarize these in vivo studies, both Klaunig and Lafferty et al. documented that acrylamide induced DNA synthesis hence cellular proliferation in testicular mesothelium in F344 rats after chronic exposure,^17^, ^18^ whereas Camacho et al. purported that acrylamide did not change the proliferation of peritesticular mesothelium.^8^ In a study in which rats take acrylamide by drinking water, of all tumours types, the only tumour incidence which significantly increased at the relatively lower acrylamide doses was scrotal mesothelioma.^19^ Consistently, Friedman et al. also confirmed acrylamide‐ ‐induced mesotheliomas of the testis.^20^ Except for those aforementioned studies, no single in vitro study related to mesothelioma or mesothelium cells exist in literature. To fill this lacuna, we aimed at examining various dose range of acrylamide on SPC212 human mesothelioma cell line, obtained from the pleural cavity of 47‐year old woman.^21^

## 2 MATERIALS AND METHODS

### 2.1 Cell culture and treatment

SPC212 mesothelioma cells were cultivated in DMEM, including 10% FBS, 2 mM of L‐glutamine and penicillin/streptomycin (100 UI per mL). The SPC212 cells was cultivated in a standard CO_2_ incubator (37°C). The cells were passaged by being dissociated with 1% trypsin/EDTA and subcultured twice each week depending on confluence, seeded at a density about 3×10^3^ cells/mL. 5 mM of acrylamide stock solution (Cat no:A9099, Sigma‐Aldrich, Missouri, US) was made with H_2_O, aliquoted and stored at −20°C. The chemical was diluted in fresh media just before the experiment.

### 2.2 MTT test

The MTT colorimetric test was employed to assess the number of live cells. The cytotoxicity reagent MTT relies on the reduction of tetrazolium dye to formazan crystals by mitochondrial succinate dehydrogenase activity.^22^ The cells were seeded into 96‐well plates at a density of 3×10^3^ cells/well, and then treated with different doses of acrylamide by serial dilution starting from 25 mM for 24 h. Afterwards, 20 μL MTT (5 mg/mL) solution was added. Three hours later the medium was disposed and the resulted formazan crystals were solubilized in 100 μL of DMSO by 10‐min shaking at room temperature. Finally, the absorbance of purple colour was measured at the wavelength of 570 nm by an ELISA reader (800TS, BioTek Instruments, Winooski, VT, US). The cell proliferation rate was calculated as follows:

(OD of the acrylamide‐treated cells−OD of blank)/(OD of the untreated cells−OD of the blank) × 100, where OD stands for optical density.

### 2.3 Neutral red uptake

Neutral red (NR) assay is a sensitive cytotoxicity test and relied on the NR dye uptake of viable cells and maintain it in the lysosomes. SPC212 cell line viability was additionally assessed employing NR assay. The 24 h before acrylamide treatment, cell seeding occurred in 96‐well culture plates with 3000 cells per well. Following a 24‐h incubation of the cells with acrylamide, they were subjected to treatment with 250 μL of a 1% NR (Neutral Red) solution for a further 3 h in humidified 5% CO_2_ atmosphere at 37°C. The NR crystals taken up by the cells were solubilized using 100 μL of dye extraction solution (49:50:1, ultrapure water: ethanol:glacial acetic acid) and assessed at 540 nm utilizing a plate reader.

The half maximal inhibitory concentration (IC50) was calculated by a linear curve drawn by using the Excel program.

### 2.4 Protein isolation

Protein isolates were used for total antioxidant capacity (TAC), total oxidant status (TOS), and oxidative stress index (OSI). Adherent SPC212 cells were treated acrylamide for 24 h, washed delicately with chilled PBS with neural pH, dislodged and centrifuged at 300–400 *g* for 15 min at 4°C in 15‐mL Falcon tubes. The pellet was suspended by cold RIPA lysis buffer (sc‐24,948, Santa Cruz Biotechnology, CA, USA), incubated for 30 min with vortexing at 5 min intervals. Thereafter, to get rid of cell debris, the supernatant was centrifuged at 16000 × *g* for 10 min at 4°C. Qubit™ Fluorometer (q33211, TMO, Waltham, MA, US) is exploited to ascertain the protein levels.

### 2.5 Total oxidant status

Total Oxidant Status (TOS) measurement was carried out by the guideline of a commercial assay kit (Rel Assay Diagnostics, Gaziantep, Turkey). In the assay, ferrous ion‐chelator complex was oxidized to ferric ion, which forms a coloured complex in acidic medium. The colour density of this complex is proportional to oxidant level in the samples. Concisely, 45 μL of sample (total protein isolate) or standard (10 μmol/L H_2_O_2_) supplied in kit or H_2_O and 300 μL of Reagent 1 were mixed, the absorbance was measured at 530 nm and recorded. Thereafter, 15 μL of Reagent 2 was added, and the absorbance was re‐measured at 660 nm after 10‐min incubation at room temperature. TOS was expressed as μmol H_2_O_2_ Equiv./L. To calculate TOS, the following formulas were used:

1. A2‐A1 = ∆Absorbance of standard or sample or H_2_O
2. TOS = ∆Absorbance of sample / ∆Absorbance of standard × 10 (concentration of standard)

### 2.6 Total antioxidant capacity

Total antioxidant capacity (TAC) measurement was carried out by the guideline of a commercial assay kit (Rel Assay Diagnostics, Gaziantep, Turkey). In the assay, 3‐ethyl‐benzothiazoline‐6‐sulfonate‐ABTS‐ is decolorized with present anti‐oxidants. Concisely, 18 μL sample (total protein isolate) or standard (1 mmol/L Trolox) supplied in kit or H_2_O and 300 μL of Reagent 1 were mixed, the absorbance was measured at 660 nm and recorded. Thereafter, 45 μL of Reagent 2 was added, and the absorbance was re‐measured at 660 nm after 10‐min incubation at room temperature. TAC was expressed as mmol Trolox Equiv./L. To calculate TAC, the following formulas were used:

3 A2‐A1 = ∆Absorbance of standard or sample or H_2_O
4 TAC = [∆Absorbance of H_2_O−∆Absorbance of sample]/[∆Absorbance of H_2_O−∆Absorbance of standard]

### 2.7 Oxidative Stress Index

The Oxidative Stress Index (OSI) represents the proportion between Total Oxidant Status (TOS) and Total Antioxidant Status (TAS). To derive the OSI, the measurement unit of TAS, denoted as Trolox H_2_O_2_ equiv. mmoles/L, underwent conversion into Trolox H_2_O_2_ equiv. μmoles/L. OSI was expressed as arbitrary unit. To calculate OSI, the following formula was used:

OSI = (TOS, μmol H_2_O_2_ Equiv./L./TAS, μmol Trolox Equiv./L) × 100.

### 2.8 Inverted microscope

The cell samples were placed on the cover glasses in a six‐well culture plate. Subsequently, they were either untreated or exposed to varying doses of acrylamide (0.39, 0.78, 1.56, 3.125, 6.25, 12.5 and 25 mM). Thereafter, an upside‐down microscope was used to examine the cellular alterations, with respect to cellular changes.

### 2.9 Haematoxylin–eosin stain

Haematoxylin–eosin stain has been a frequently‐employed, principal technique and helps display a broad range of cytoplasmic, nuclear, and extracellular matrix features.^23^ The morphological changes of acrylamide‐treated cells were scrutinized by Haematoxylin–eosin staining method. SPC212 cells were seeded into six well plates at a density of 3 × 10^5^ cells/well. After sufficient incubation, the cells were washed with PBS and allowed to 24‐h incuabation with different doses of acrylamide. Ensuingly, the cells underwent fixation using methanol kept at a low temperature. Then, the cells were washed and stained with haematoxylin for 3 min and submerged into 1% of ammonia. Followingly, the cells were stained with eosin stain (1–2 min). Finally, the cell samples were rinsed with H_2_O, left to dry and closed onto slide with a water‐based medium.

### 2.10 May Grunwald staining

The appearance of the acrylamide applied SPC212 cell line was scrutinized upon staining with a tweaked version of May‐Grunwald‐Giemsa staining in the article mentioned.^24^ SPC cells were seeded on cover glasses and rinsed three times with 1‐min intervals, and followed with a chilled methanol fixation for 10 min, then re‐rinsed. Thereafter, cover glasses were submerged into MayGrunwald solution for 2 min, then the solution was discarded and cover glasses were rinsed with pure H_2_O and stained with 50% Giemsa solution for 5 min. After 3 PBS distilled water washes, the aqueous mounting medium was added on a slide, onto which cover glasses were covered. Finally, the cell samples were scrutinized under a light microscope.

### 2.11 Acridine orange/ethidium bromide staining

To perform acridine orange/ethidium bromide (AO/EB) (Sigma, St. Louis, MO) staining, the cells were seeded at a density of 3 × 10^5^ cells per well onto six‐well plates and incubated with or without different doses of acrylamide for 24 h after they adhered. At the end of the incubation time, cells were harvested, resuspended in a fresh culture medium, and then stained with acridine orange/ethidium bromide (1:1, 0.1 mg/mL, Sigma) solution. Eventually, 10 μL of cell suspension stained with AO/EB solution was immediately observed with a confocal microscope (Zeiss, Germany).

### 2.12 Visualizing cell nuclei with DAPI fluorescence

For 4′,6′‐diamidino‐2‐phenylindole hydrochloride (DAPI) staining, the cells were seeded at a density of 3 × 10^5^ cells per well onto six‐well plates and incubated with or without acrylamide (2.65 mM) for 24 h. At the end of the incubation time, the cells were fixed by 100% ice‐cold methanol for 10 min. Next, 15‐min incubation with DAPI in dark was carried out on the samples. Eventually, the cover glasses were adhered onto slides with an aqueous‐based mounting medium and visualized through a fluorescence‐attached microscope.

### 2.13 Immunocytochemistry (IM)

300,000 cells of SPC212 were seeded on six‐well culture plate and 2.00 mM of acrylamide was applied for 24 h. Afterwards, the cells were fixed with 100% ice‐cold methanol for 10 min, following three washing steps. Then, the 10 min‐blocking solution was applied, which was followed by three washing steps. Following this, the cells were subjected to an overnight incubation at 4°C with primary antibodies against Bax (from SCBT, CA, US), Bcl‐2 (from SCBT, CA, USA), and PCNA (from Bioss Antibodies Inc., MA, US), with sufficient humidity provided. On the following day, biotinylated goat anti‐polyvalent and streptavidin‐peroxidase reagents were consecutively introduced, each for a duration of 10 min. AEC chromogen is used to produce red colour images, while haematoxylin was used for counterstaining. Finally, the samples were applied a water‐based mounting medium and examined through a light microscope.

### 2.14 Statistics

MTT and NR assay data were analysed using GraphPad Prism version 7.00 for Windows (GraphPad Software, La Jolla California USA). All data followed Gaussian distribution. Therefore analysed with One‐way ANOVA ensued by Dunnett's post hoc test. Data were provided with average + standard deviation. *p* < 0.05 was accepted as statistically significant.

## 3 RESULTS

### 3.1 Findings on cellular toxicity

**FIGURE 1:**
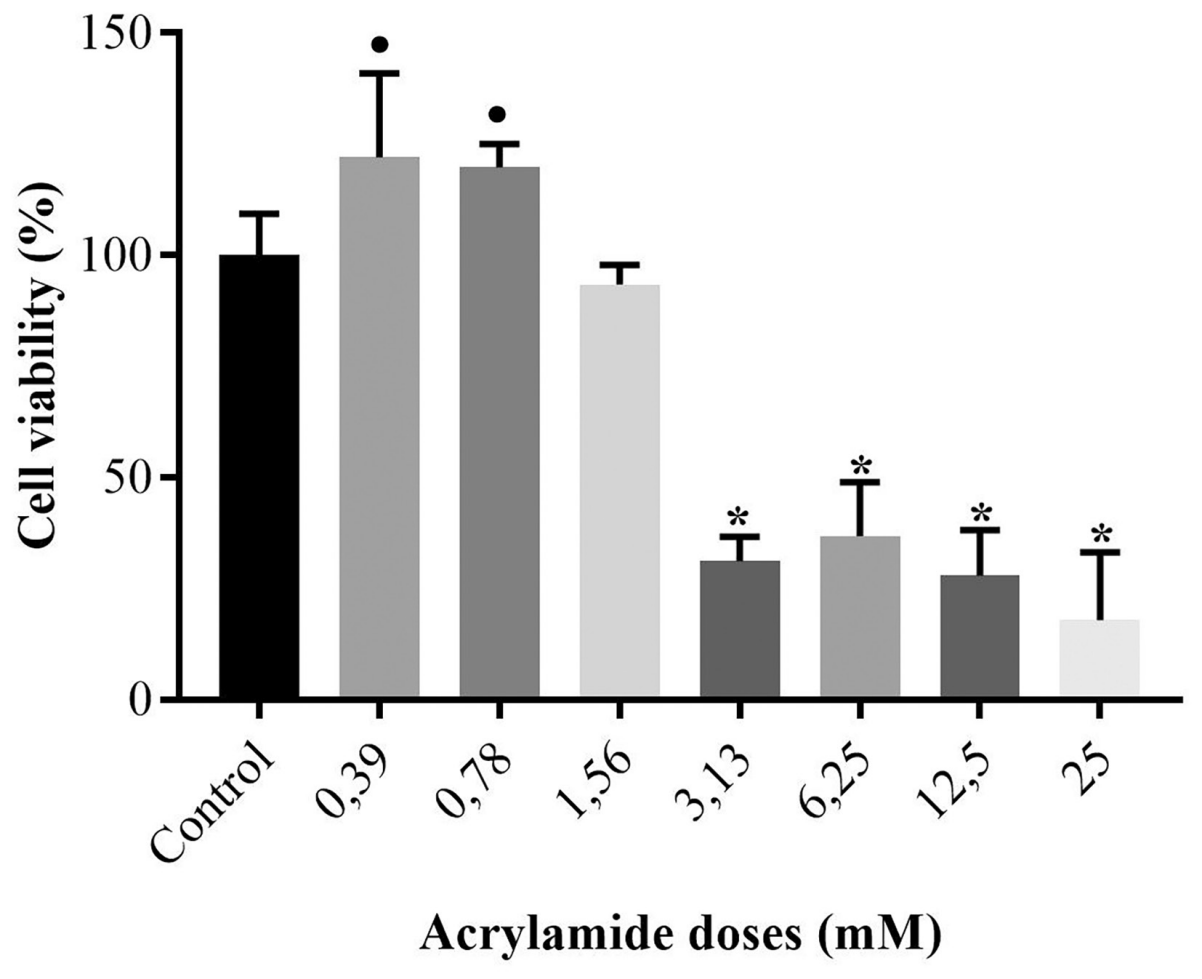
Cell viability percentages versus different acrylamide doses based on MTT test findings. • and * represent significant distinction with *p*‐values less than 0.05 and 0.001, respectively, in comparison to the untreated cells.

**FIGURE 2:**
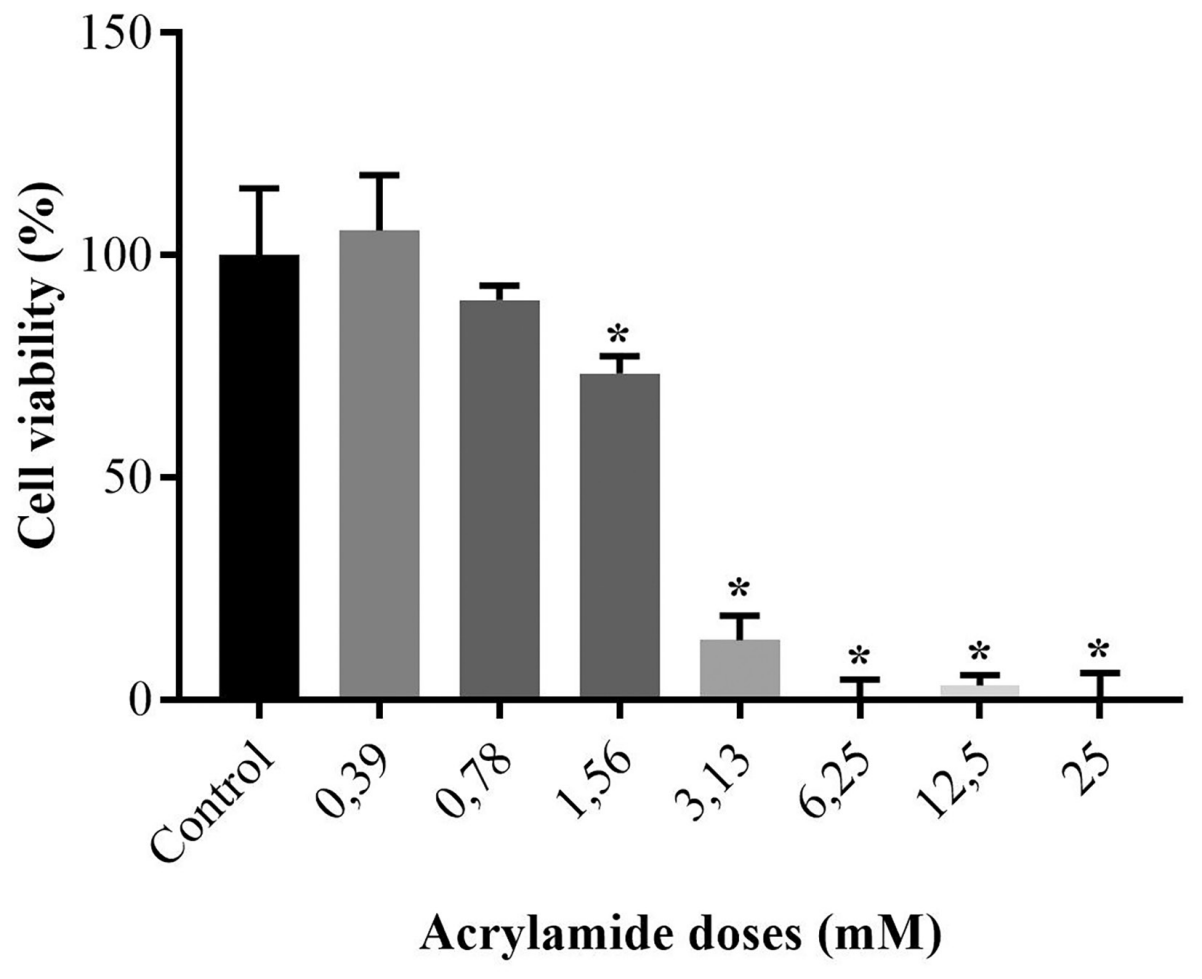
The percentages of live cells versus various acrylamide doses based on Neutral red test findings. * represents significant distinction with *p*‐values less than 0.001 in comparison to the untreated cells.

The cytotoxic effect of acrylamide on the growth of SPC212 cells was measured using the MTT and NR uptake test. The percentages of acrylamide‐treated cell viabilities were calculated relative to control cell viability, shown in Figures 1, 2 and Table 1.

**TABLE 1:** Numerical values and respective *p* values of MTT and neutral red test results as percentage.

Acrylamide doses (mM)	MTT cell viability (%) (mean ± SD)	Neutral red cell viability (%) (mean ± SD)	*p* values
Untreated	100.0 ± 9.3	100.0 ± 15.1	—
0.39	122.1 ± 18.9	105.6 ± 12.4	<0.01 •
0.78	119.8 ± 5.3	89.9 ± 3.3	<0.01•
1.56	93.5 ± 4.4	73.4 ± 3.9	<0.001 (#)
3.13	31.2 ± 5.5	13.5 ± 5.5	<0.001 (*)
6.25	36.8 ± 12.2	−0.2 ± 4.5	<0.001 (*)
12.50	27.9 ± 10.2	3.2 ± 2.3	<0.001 (*)
25.00	18.0 ± 15.2	−3.0 ± 8.3	<0.001 (*)

*Note*: Significant difference according to both (*), only MTT (•) and only neutral red (#) assays.

According to MTT results, at lower acrylamide doses of 0.39 and 0.78 mM, the cell viabilities increased to 122.1 and 119.8%, respectively and showed a significant difference (both *p* < 0.01) from the viability of untreated cells. 1.56 mM of acrylamide was not significantly different than untreated cells, although viability decreased to 93.5%. At the doses of 3.13, 6.25 and 12.50, acrylamide toxicity was evident, the cell viabilities decreased to almost 30% (all *p* < 0.001 vs. untreated cells). 25 mM of acrylamide pull the percentage of live cells down to 18% (*p* < 0.001 vs. untreated cells).

As to NR results, at the lowest two doses (0.39 and 0.78 mM), the cell viabilities of the cells were 105.6 and 89.9% (*p* > 0.05 vs. untreated cells). 1.56 mM of acrylamide lowered the percentage of live cells to 73.4% (*p* < 0.001 vs. untreated cells). At 3.13 mM, the percentage of live cells further lowered to 13.5% (*p* < 0.001 vs. untreated cells). Finally, regarding three highest concentrations, there were almost no viabilities following 24 h acrylamide treatment.

The acrylamide concentration utilized in upcoming assays were determined based off of cytotoxicity assays' findings. IC50 and IC75 concentrations were determined respectively as 2.65 and 3.00 mM. In H‐E, DAPI, May Grunwald and AO/EB stainings, IC50 and IC75 concentrations were exploited. In IHC, 2.00 mM, a dose smaller than IC50, was preferred to detect the levels of apoptotic proteins prior to a complete cell death.

### 3.2 Oxidative stress results

#### 3.2.1 Total oxidant status

**FIGURE 3:**
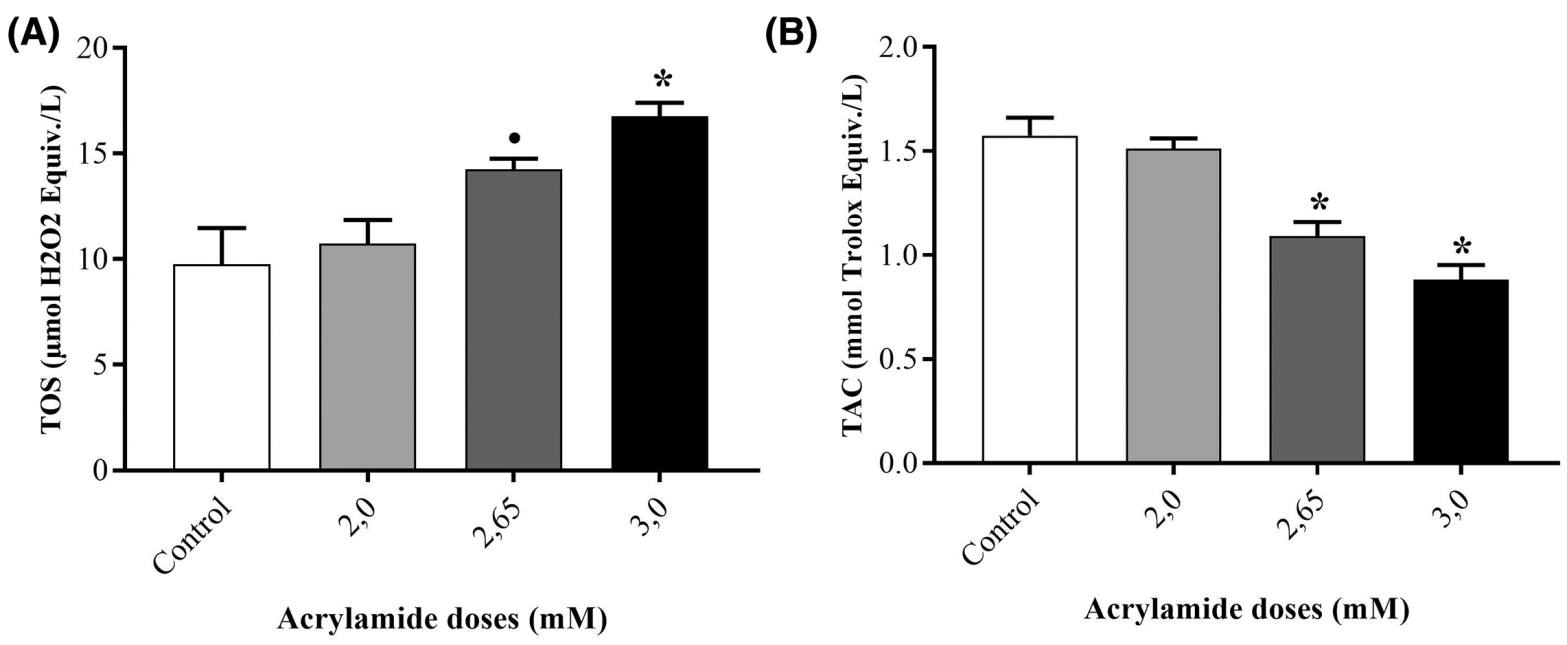
The measurement of whole oxidants of the SPC212 cells in (A) and whole antioxidants in (B). Upon acrylamide‐treatment. • and * represent significant distinction with *p*‐values less than 0.05 and 0.001, respectively, in comparison to the untreated cells.

In TOS analysis (Figure 3A), there was a dose‐dependent increase. At the dose of 2.00 mM, there was no significant difference (*p* > 0.05 vs. control). However, 2.65 and 3.00 mM of acrylamide increased TOS levels almost 1.5 (*p* > 0.05) and 1.7 (*p* > 0.001) folds when compared to control.

#### 3.2.2 Total antioxidant capacity

In TAC analysis (Figure 3B), there was a dose‐dependent decrease. At the dose of 2.00 mM, there was no significant difference (*p* > 0.05 vs. control). However, 2.65 and 3.00 mM of acrylamide decreased TAC levels almost by %30 and %44 (both *p* > 0.001) folds relative to untreated cells.

#### 3.2.3 Oxidative stress index

**FIGURE 4:**
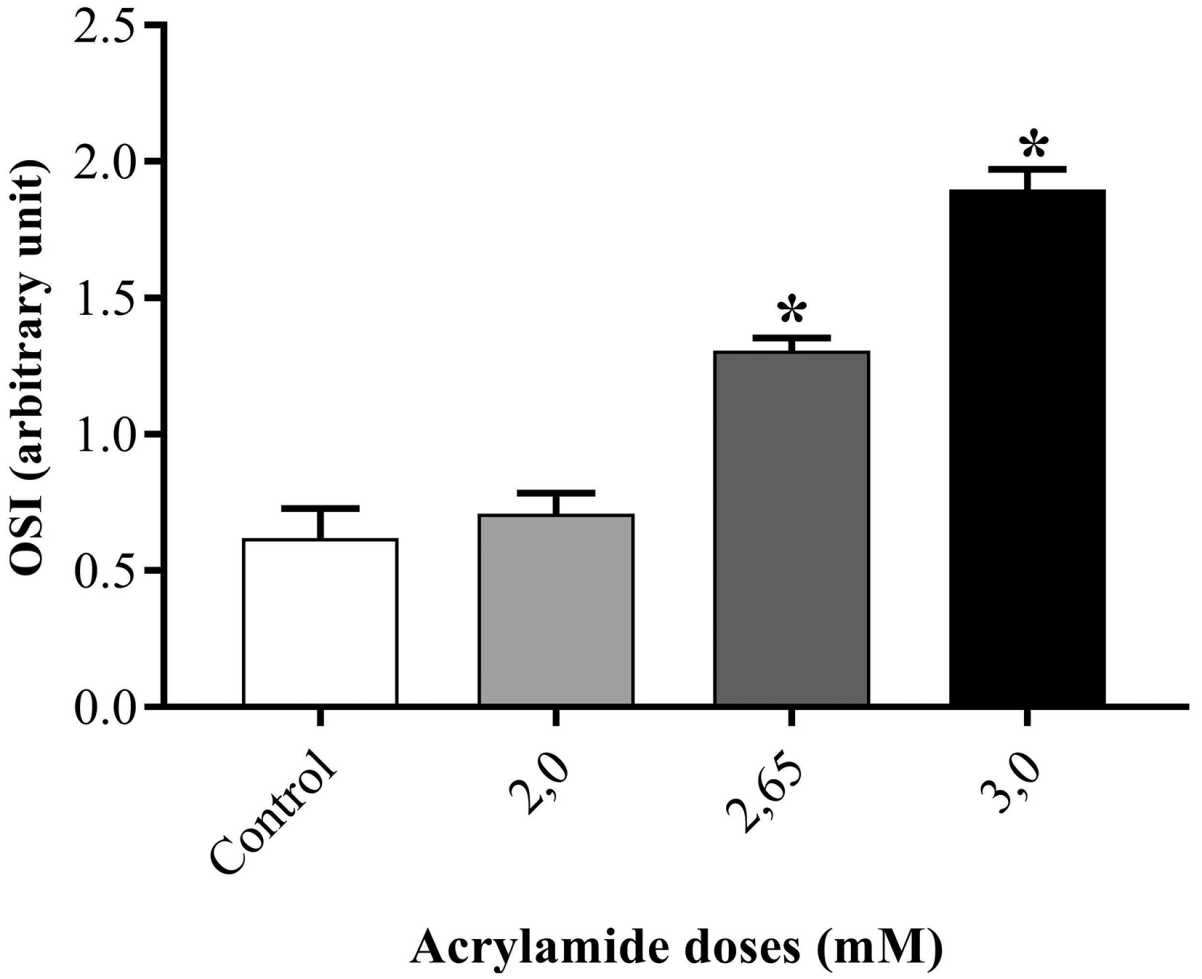
The index of oxidative stress of acrylamide‐applied SPC212 cell line. * represent significant distinction with *p*‐values less than 0.001 in comparison to the untreated cells.

In OSI results (Figure 4), 2.00 mM of acrylamide exhibited no significant difference (*p* > 0.05 vs. untreated cells). At the doses of 2.65 and 3.00 mM, an almost 2.0 and 3.0‐fold increases (both *p* > 0.001 vs. control) were detected in OSI levels.

### 3.3 Morphology analysis results

#### 3.3.1 Inverted microscope

**FIGURE 5:**
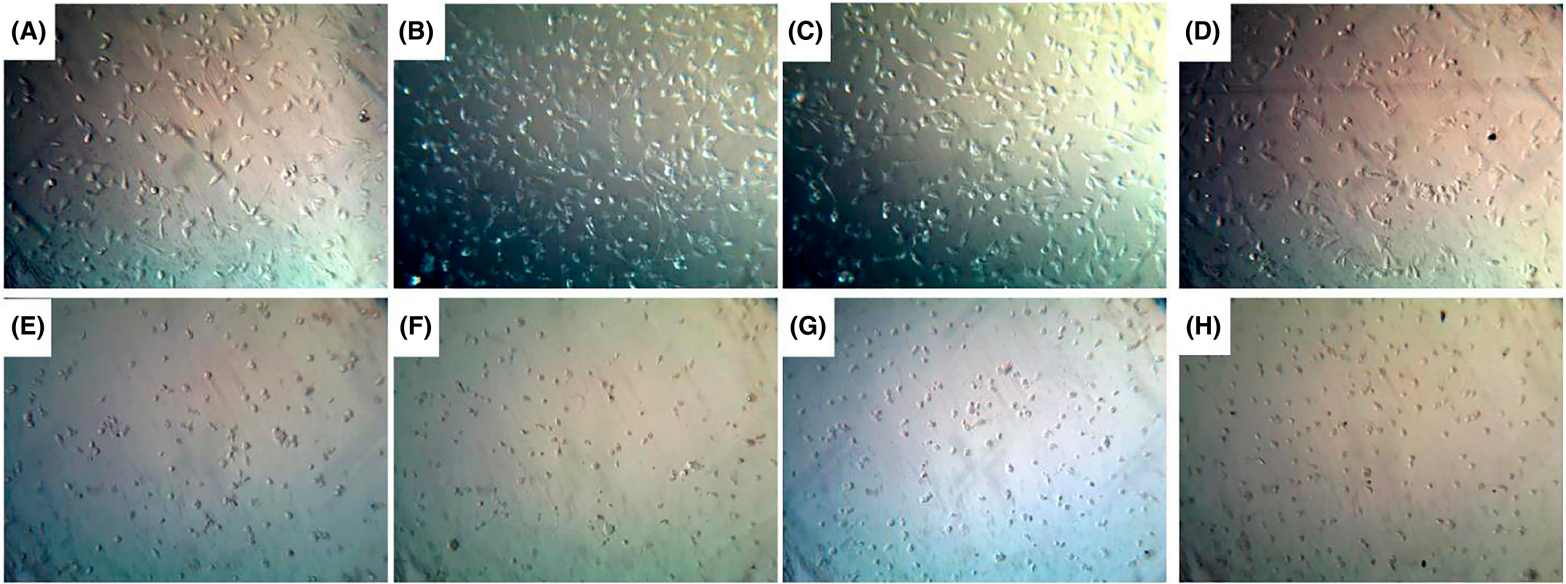
The pictures of SPC212 cell line upon acrylamide administration. (A) Control cell line, (B) 0.39, (C) 0.78, (D) 1.56, (E) 3.125, (F) 6.25, (G) 12.5 and (H) 25 mM of acrylamide treated cells. The increased cell proliferation in B and C and increased cell inhibition in (E–H) are evident.

Under the inverted microscope (Figure 5), 0.39 and 0.78 mM of acrylamide groups demonstrated a cellular proliferation. At the dose of 1.56 mM, cellular density re‐decreased. At the dose of 3.125 mM and above, apparent cellular degenerations including cellular shrinkage were observable.

#### 3.3.2 Light microscope

**FIGURE 6:**
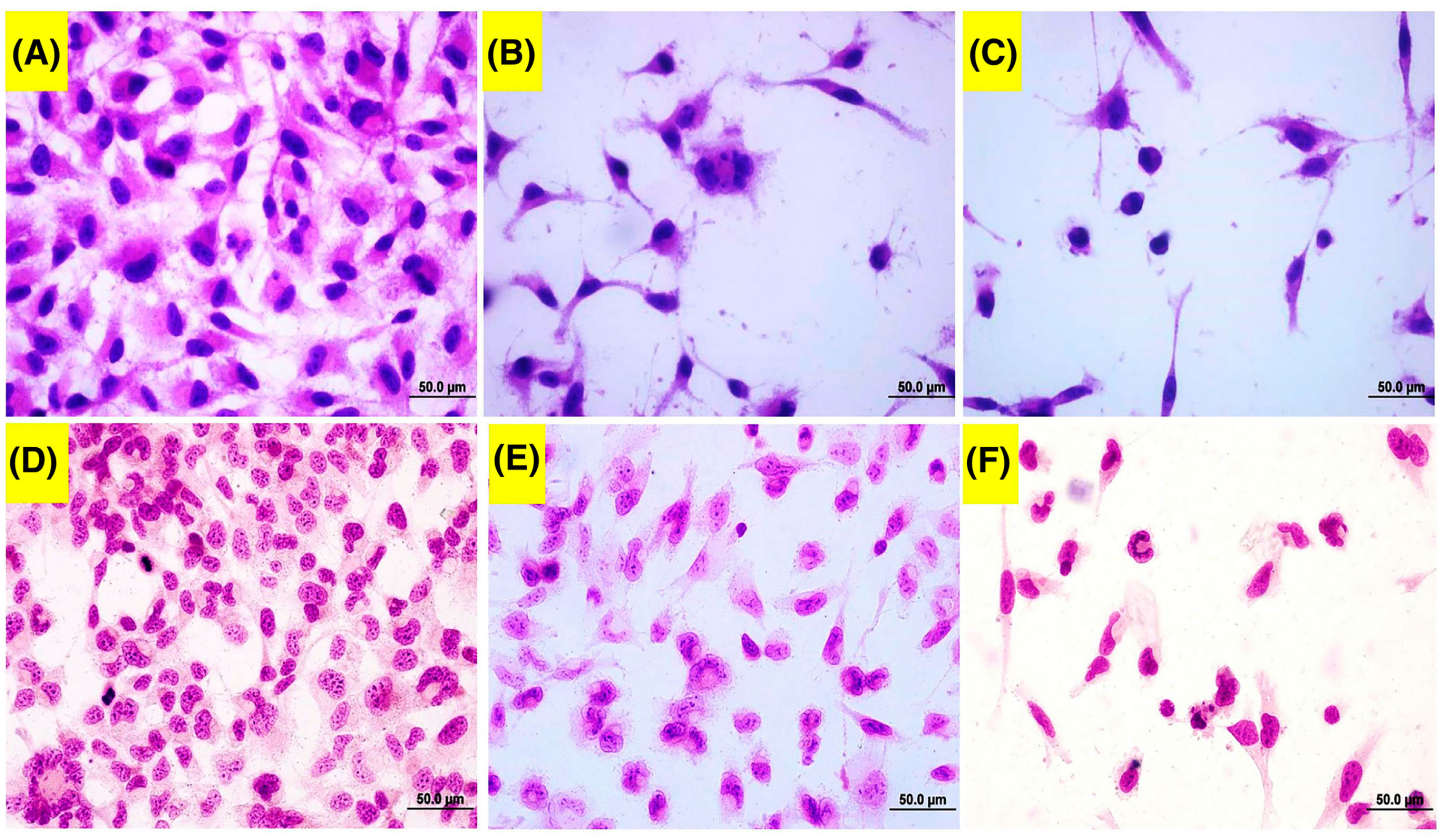
Haematoxylin–eosin and May Grunwald images of acrylamide applied SPC212 cells. (A and D) Control cells, (B and E) 2.65 mM, and (C and F) 3.00 mM of acrylamide‐applied cells. (A, B) indicate 100 μm, while the others indicate 20 μm.

In Haematoxylin–eosin staining (Figure 6A–C) and May Grunvald (Figure 6D–F), untreated cells possessed a typical cytoplasm and nucleus with their standard size. However, acrylamide‐treated cells demonstrated several abnormalities including the condensed and kidney‐shaped nucleus, cellular shrinkage, membrane blebbings and apoptotic bodies.

#### 3.3.3 Confocal microscope

**FIGURE 7:**
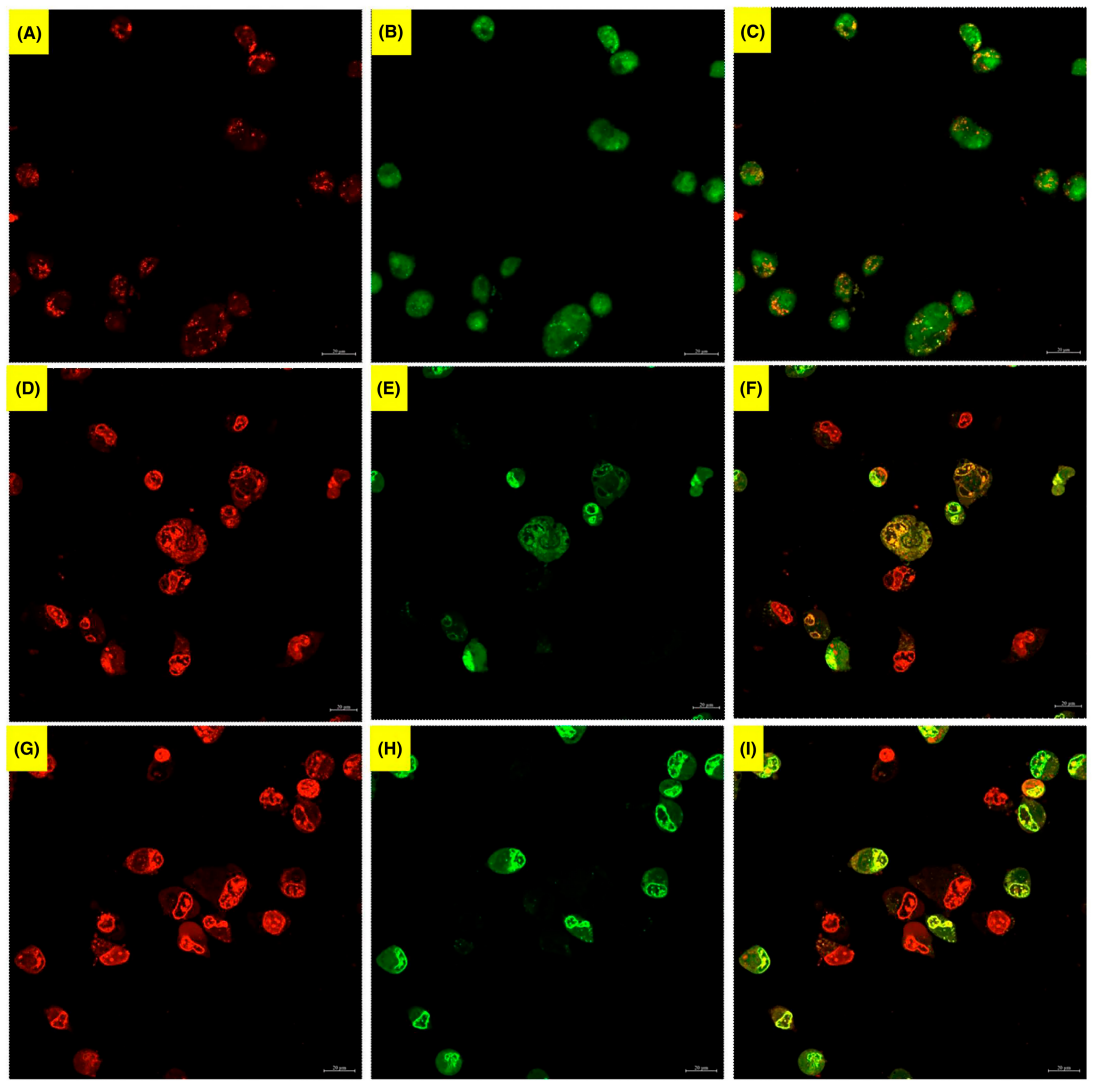
Fluorescent microscopy of SPC cells stained with acridine orange/ethidium bromide, (A‐C) Untreated cells. (D‐F) 2.65 mM and (G‐I) 3.00 mM of acrylamide‐8 treated cells.

To uphold if the mode of cell death is apoptosis, AO/EB flourescent staining was carried out. Acridine orange (AO) can penetrate into both viable and dead cells, while EB only enters the cells, of which membrane integrity is about to deteriorate. Our AO/EB staining is depicted in Figure 7. In the untreated cells, the nuclei of cells were commonly green, but not all were homogenous green, there were some orange‐like staining in the cytoplasm of certain cells. We think this orange‐like colour was because of stained RNAs in the cytoplasm of the cells. In 2.65 mM of the acrylamide‐treated group, the cells with red‐stained nuclei were common. In addition, there were cells with yellow nuclei as well as orange‐coloured swollen cells. In 3.0 mM of the acrylamide‐treated group, the same cellular degenerations observed at 2.65 mM dose persisted. In both acrylamide group, the cells' nuclei were eccentric, condensed and crescent‐shaped (Figure 7).

#### 3.3.4 Fluorescent microscope

**FIGURE 8:**
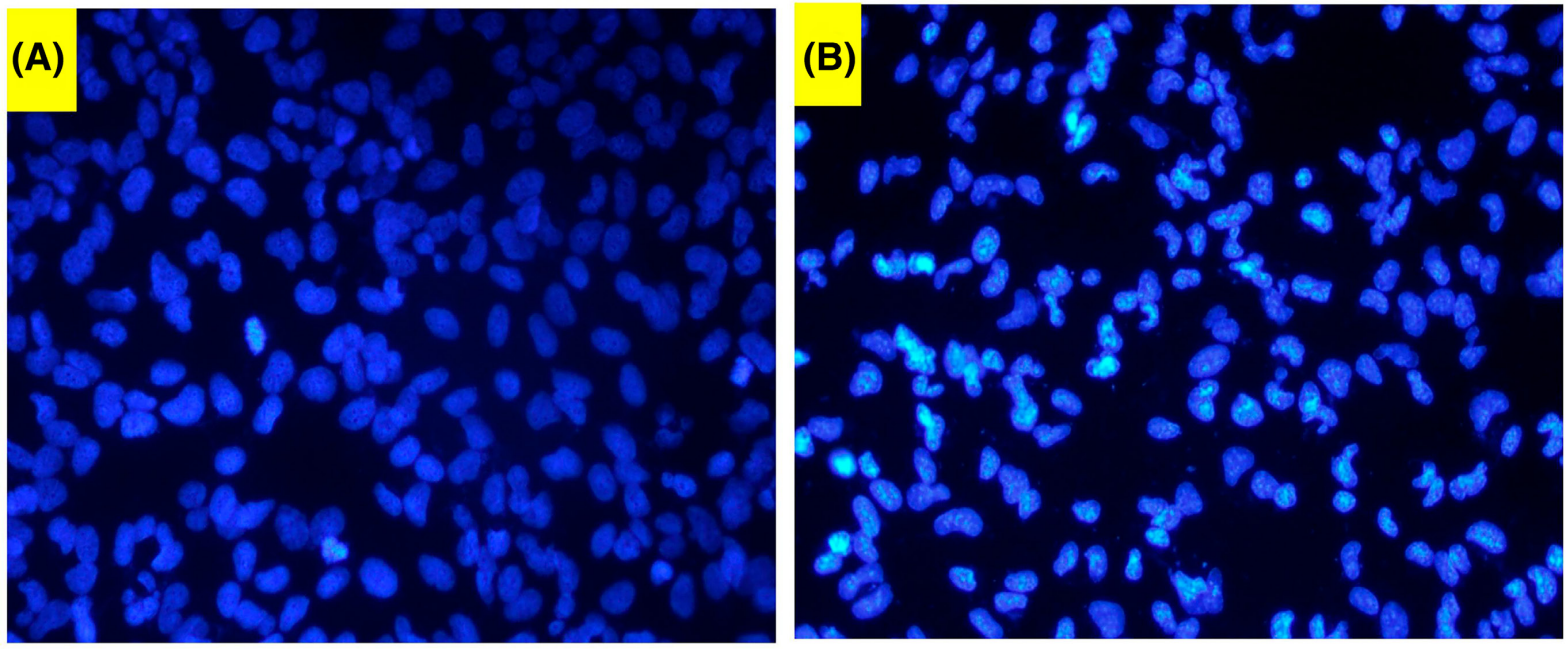
DAPI images of SPC cells. (A) Untreated and (B) 2.65 of acrylamide treated cells.

DAPI is an extensively used fluorescent dye that binds DNA by transiting cell membrane. DAPI stained sections are depicted in Figure 8. The nuclei of acrylamide untreated cells had characteristic shape and morphology. However, acrylamide‐applied group possessed nucleus deformations like fragmented and condensed nuclei.

### 3.4 Immunocytochemistry results

**FIGURE 9:**
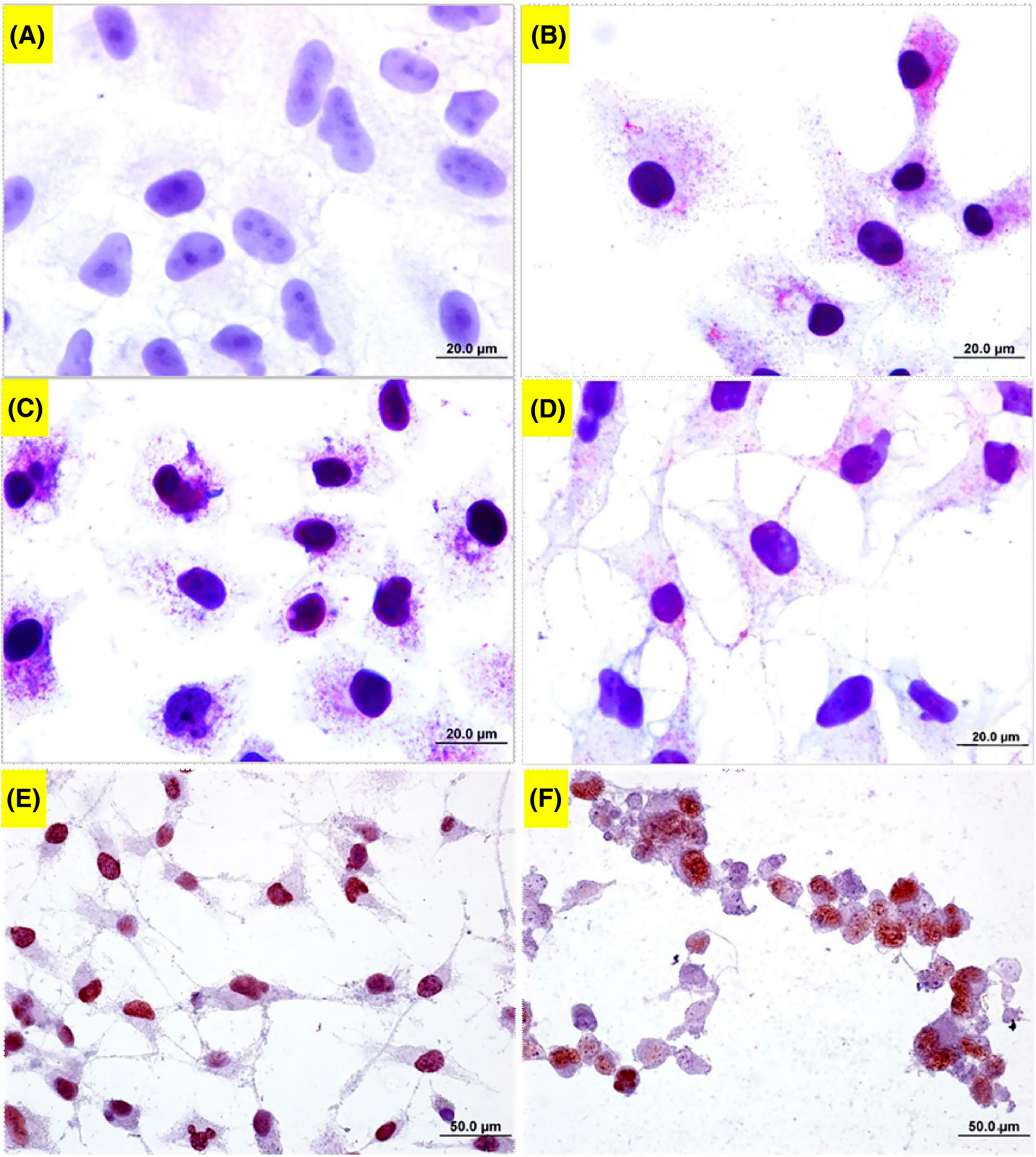
Bax/Bcl‐2 stains of acrylamide‐applied SPC212 cell line. (A) Bax staining of untreated SPC cells. (B) Bax staining of 2.00 mM of acrylamide‐treated SPC cells. (C) Bcl2 stain of control SPC cells. (D) Bcl2 stain of 2.00 mM of acrylamide‐treated SPC cells. All bars show 20 μm. (E) PCNA stain of untreated SPC cell line. (F) PCNA staining of 2.00 mM of acrylamide treated SPC cells. The distances on the scale bars from (A to D) correspond to 20 μm, and for (E, F), they correspond to 50 μm.

Bax, Bcl‐2 and PCNA immunostainings are depicted in Figure 9. Untreated SPC212 cells displayed negative staining. The density of Bax immunopositive cells increased in 2.0 mM of acrylamide treated cells. As for the Bcl‐2 staining, untreated SPC212 cells were immune‐positive. The cells were barely stained in 2.00 mM of acrylamide treated cells. On the other hand, nearly all cells were stained in untreated cells in PCNA staining. However, there were cells with unstained nuclei in acrylamide‐treated cells (Figure 9).

## 4 DISCUSSION

In the present study, for the first time, a mesothelioma cell line was used in an acrylamide toxicity study. Herein, we observed a dose‐dependent antiproliferative effect of acrylamide starting from 1.56 mM of acrylamide. We reported IC50 value of acrylamide for SPC212 cells as 2.65 mM. We also observed apoptotic signs in our morphological analyses. Intriguingly, we observed a proliferation‐inducing tendency of acrylamide at lower doses (0.39 and 0.78 mM). Instead of ignoring this, we find it worthwhile to mention in the discussion.

In the cytotoxicity assay, the IC50 of acrylamide against SPC212 cells was 2.65 mM based on MTT test. For the comparison of our results with those in previous studies, either we used reported IC50 values or that we calculated the IC50 value from MTT data in the studies that IC50 was not given. To compare our previous results with this current one, we had revealed IC50 of acrylamide against A549 lung^11^ and NIH/3 T3 fibroblast cells^12^ as 4.6 and 6.73 via MTT test, which are 1.7 and 2.5 times the IC50 against SPC212 cells. As such, it is plausible to say that SPC212 mesothelioma cells are more sensitive to acrylamide than those cells. Other IC50s of acrylamide reported belongs to the PC12 pheochromocytoma^25^ and HepG2 hepatocarcinoma^26^ cells, which are 5.0 and 7.18 mM, respectively. Likewise, both IC50 are almost 1.9 and 2.7 times the IC50 of present study. In the other studies with Caco‐2 colon^27^ and IEC‐6 intestine^28^ cells, IC50s of acrylamide were estimated to be similar and around 5 mM. Besides, in one study, IC50 against SH‐SY5Y neuroblastoma cells was found higher than 5 mM, the highest dose used in the study.^29^ The IC50 of acrylamide against another neuroblastoma cell line, NB‐1^30^ and primary lymphocytes^31^ was estimated as 144 ug/mL and 459.7 mg/L equaling to 2.03 and 6.57 mM, respectively. Finally, in a study with BV2 microglial cells,^32^ IC50 of acrylamide, was calculated to be roughly 3.3 mM from the MTT data of study. Altogether, except NB‐1 cells, all aforementioned cells mentioned possessed higher IC50 of acrylamide than ours, SPC212 cells.

Cell death is fated when no longer survival signals of cell overcome the death‐ inducing ones. When the cell faces with different burdens, it utilizes various courses to handle with it such as using existed antioxidants, fueling antioxidant synthesis, trying to phagocyte it as well as informing others cells and producing radicals. When the loads and oxidants borne by the cell overwhelmed its capacity, the cell undergoes to death. There are various types of cell death that discovered before and recently. Still, new cell death mechanisms are ongoing. Of them, apoptosis is the programmed cell death with known characteristics, which renders it to be distinguished better than others.^33^ Apoptotic effect of acrylamide was shown in several studies on different cell types including lung,^11^ fibroblasts,^12^ microglial^32^ cells but this study is the first to show it on mesothelioma cells. To see apoptotic effect of acrylamide on mesothelioma cells, we examined morphology of the cells in terms of apoptotic hallmarks; besides, we stained the cells with pro‐ and anti‐apoptotic marker proteins. AO emits a green fluorescence if it transits the cell membrane and intercalates into DNA whereas EB binds nuclear DNA of damaged cells and emit a red/orange fluorescence. In literature, in two studies done by the same groups, Liu et al. (2015) and Song et al. (2017) used AO‐EB staining for acrylamide‐treated BV2 microglial cells. In the former study,^32^ 0.5, 1 and 2 mM of acrylamide was applied. In a dose dependent manner, number of red/orange coloured cells increased. In the latter study,^34^ only 2 mM of acrylamide was used, and almost all control cells emitted green fluorescent, whereas in 2 mM of acrylamide treated cells, the orange fluorescent was dominant. Similarly, present research displayed a concentration‐dependent decrease in green coloured cells and increase orange and bright yellow‐ coloured cells. In both study, an increase in Bax and a decrease in Bcl‐2 was detected. In their DAPI staining, they recorded nuclear condensations after acrylamide treatment. Yu et al. (2019) administered acrylamide orally to pregnant mice at gestational phases and detected proliferation‐inhibiting and apoptosis‐inducing effect of acrylamide in the placenta. In this study, while Ki65 and Bcl‐2 decreased, apoptosis‐related proteins Bax, cleaved caspase 3 and 8 increased.^35^ In the study of Kacar et al. (2018), acrylamide was reported to increase Bax and decrease in PCNA expression in testis tissue.^9^ In our study, consistent to both studies, we also detected a decrease in PCNA and Bcl‐2 and an increase in Bax protein.

In our study, we found that acrylamide increased the proliferation of mesothelioma cells at the doses of 0.39 and 0.78 mM in the MTT test. Although we did not perform further proliferation assay, we also confirmed this with images of inverted microscope. Xu et al. (2017) purported that the doses of acrylamide lower than 100 μM induced proliferation in HepG2 cells, substantiated by both MTT and EdU assays.^36^ Our putative proliferation‐ inducing doses in terms of micromolar levels, are 390 and 780 μM, which are almost 4 and 8 folds greater than the reported dose of Xu et al. Maybe the lower acrylamide doses than 390 μM will also exert proliferative action on SPC cells. Shan et al. (2014) also exhibited that lower concentration of acrylamide (≤100 μM) prominently provoked the proliferation of HepG2 cells but not other cell lines (MDA‐231, HeLa, A549, and PC‐3). Also, this amount of acrylamide activated CYP2E1 expression, which is the enzyme converting acrylamide into a more reactive chemical, glycidamide.^37^ In the same study, knockdown of this enzyme abated the proliferating effect of acrylamide. Besides, when we check the Edu assay results of this study, also the acrylamide dose of 500 μM for 24 h was apparently proliferative. What's more, Brdu incorporation assay in the study Lee et al. (2019) conducted on 3 T3‐L1 cells showed that acrylamide doses of almost 70, 140 and 700 μM (converted from ng/mL unit) did not proliferate the cells, on the contrary, exerted cytotoxic effect on these cells.^38^ In conclusion, acrylamide exerts a strong proliferative effect on certain cells while affecting others in a different way. For our used cell line, SPC212, it exerted proliferative effects at low doses, but inhibiting effects on relatively high doses.

Herein, we investigated the in vitro effect of acrylamide on SPC212 mesothelioma cell line. We found anti‐proliferative and apoptotic effects of acrylamide with a number of morphological analysis as well as two different cytotoxicity tests. This is the first study searching effect of acrylamide in vitro on SPC212 cells. To note, we also detected cellular proliferation at lower doses. As such, apart from acrylamide's toxic effect, we also strongly suggest the scientific community to give sufficient importance to the lower doses of acrylamide, considering the fact that most of us expose acrylamide at lower levels in daily life.

## AUTHOR CONTRIBUTIONS

**Sedat Kacar:** Conceptualization (lead); data curation (lead); formal analysis (lead); investigation (lead); methodology (lead); project administration (lead); visualization (lead); writing – original draft (lead); writing – review and editing (lead). **Ozlem Tomsuk:** Conceptualization (supporting); data curation (supporting); formal analysis (supporting); investigation (supporting); methodology (supporting); writing – review and editing (supporting).

## CONFLICT OF INTEREST STATEMENT

The authors declare no conflicts of interest related to this study.

## Data Availability

None.
